# Scientific Items

**Published:** 1881-01-20

**Authors:** 


### SCIENTIFIC ITEMS.

**The Prall System of Heating** —During their recent convention in this city the members of the American Society of Civil Engineers were entertained by the Prall Union Heating Company. The dinner was cooked throughout by superheated water; and whatever may have been the cost on the relative economy of the system, the cooking was accepted as unquestionably satisfactory.

That bread can be baked and meat roasted by hot water may seem quite incredible to those who think of boiling water only as commonly seen in open vessels. Under atmospheric pressure water can be heated no higher than 212°, far below a roasting temperature. But when confined there is no limit to the temperature it may receive save the weakness or strength of the containing vessel.

The Union Heating Company propose to supply heat and power to houses by a system of pipes circulating water heated under pressure to about 376°, that is, a pressure of about 160 pounds above the atmosphere. In being conveyed a mile in boxed pipes, under ground, the water, it is claimed, loses not more than 1°, so that a temperature of 375° can be maintained in the pipes of a cooking range, a heat sufficient for all culinary purposes. The heating of houses can be effected either by air currents circulating around hot-water coils, or by means of steam radiators, the hot water being converted into steam in small converting chambers.

In the operation of the system, central boiler stations will be established in districts of about one square mile area. The pipes conveying the superheated water from the central station and back again, are laid in the same trench, and are so connected as to allow a forced circulation. The return pipe conveys to the generator all the water not drawn off for domestic or other purposes, thereby saving all the heat not available for heating purposes or for steam power.—*Scientific Am.*

**The Utilizing of the Tide.**—A Philadelphia engineer has invented, it is claimed, a machine by which the power of the tide can be utilized. Numerous plans have been proposed for the accomplishment of this most desirable end, but only under exceptional conditions have they been practical or economical. If the new device can harness the tide in an open channel, so as to convert any considerable portion of the vast power into working force, the inventor will rank among the great benefactors of humanity. Emerson says somewhere: "Hitch your wagon to a star." A device for utilizing mechanically the free tides, as they sweep along our shores, would come next to that, since it would enable us, through converters and electricity, to hitch our wagons to the sun and moon.—*Scientific American.*

**Where our Forests are Going.**—To make shoe pegs enough for American use consumes annually 100,000 cords of timber, and to make our lucifer matches, 300,000 cubic feet of the best pine are required every year. Lasts and boot-trees take 500,000 cords of birch, beech and maple, and the handles of tools 500,000 more. The baking of our bricks consumes 2,000,000 cords of wood, or what would cover with forest about 50,000 acres of land. Telegraph poles already up represent 800,000 trees, and their annual repair consumes about 300,000 more, The ties of our railroads consume annually thirty years' growth of 75,000 acres, and to fence all our railroads would cost $45,000,000, with a yearly expenditure of $15,000,000 for repairs. These are some of the ways in which American forests are going. There are others; our packing boxes, for instance, cost, in 1874, $12,000,000, while the timber each year in making wagons and agricultural implements is valued at more than $100,000,000.—*Fishkill Standard.*

**The Population of the Earth.**—Boehm and Wagner calculate and show the population of the world to be very near fourteen hundred and fifty-six millions of people, and nearly seventeen millions more than it was at the time of the last issue of their publication, nineteen months ago. It seems rather startling, at first sight, to find that the population of the earth is increasing at the rate of *nearly a million persons per month*; but a little consideration shows that this is quite possible, since the rate of increase of population in most countries, of which we have trustworthy statistics, exceeds one per cent. per annum. Asia is said to contain considerably more than half the population of the globe, or eight hundred and thirty-five millions; Europe, three hundred and sixteen millions; Africa, two hundred and six millions; America, ninety-five millions; Australia and Polynesia, four millions. Bearing in mind the different areas of the continents, we can see that America will long be able to absorb, to the advantage of itself and of all other nations, the surplus population of the rest of the world, even if it should exceed twelve millions per annum.—*Boston Journal of Chemistry.*

**Transforming Sound into Light.**—Mr. Treve has described to the French Academy of Sciences an experiment with an apparatus which he calls a singing condenser, by which he believes he effects the transformation of sound into light. When a current of electricity is brought to bear upon his condenser a sound is produced, which he attributes to the vibrations of the air in the condenser, produced by the shock of the electric current. Reversing this experiment, he placed the condenser in a Geissler tube, and brought the two poles of the electric current to bear upon the condenser through the electrodes of the table. The tube was then connected with an air pump. The condenser sounded as usual when the current was directed to it under the ordinary atmospheric pressure; but when the air was withdrawn the sound became more and more feeble, until, as a vacuum was produced, it ceased entirely, and a clear, bright light appeared, sparkling like pearls, from the leaves of the condenser—quite unlike the ordinary pale, vague light of the Geissler tubes.—*Boston Journal of Chemistry.*

**Tenacity of Iron.**—Recent experiments by Piazzoli appear to establish the fact that the tenacity of iron increases on magnetization.

